# Circle of Willis Configuration and Thrombus Localization Impact on Ischemic Stroke Patient Outcomes: A Systematic Review

**DOI:** 10.3390/medicina59122115

**Published:** 2023-12-03

**Authors:** Audrius Širvinskas, Givi Lengvenis, Giedrius Ledas, Valerija Mosenko, Saulius Lukoševičius

**Affiliations:** 1 Medical Faculty, Vilnius University, M. K. Čiurlionio g. 21, LT-03101 Vilnius, Lithuania; audrius_sirvinskas@yahoo.com (A.Š.); giedriusledas@gmail.com (G.L.); 2 Republican Vilnius University Hospital, Šiltnamių g. 29, LT-04130 Vilnius, Lithuania; 3 Medical Academy, Lithuanian University of Health Sciences, LT-44307 Kaunas, Lithuania

**Keywords:** anatomic variants, circle of Willis, stroke

## Abstract

*Background and Objectives*: The published literature highlights the fact that the integrity of the Circle of Willis has a direct impact on stroke outcome, especially in cases of distal internal carotid T occlusion. The aim of this study was to review the available data on the Circle of Willis configuration and thrombus location impact on patient outcome in cases of ischemic stroke. *Materials and Methods*: A systematic search according to PRISMA guidelines was performed in PubMed, Cochrane, and EMBASE databases to identify studies investigating the influence of Circle of Willis variants on ischemic stroke outcomes published up to March 2023. The manuscripts were reviewed by three researchers separately and scored on the quality of the research using the MINORS criteria. *Results*: After screening 157 manuscripts, 11 studies (*n* = 4643) were included. Circle of Willis integrity plays a vital role in stroke outcome, especially when T-form occlusions are present. Despite this, in the event of M1 occlusion Circle of Willis configuration does not play an important role. In cases of distal internal carotid artery occlusion, the presence of a fully developed contralateral A1 segment and anterior communicating artery is essential for a favorable stroke outcome. *Conclusions*: The preserved integrity of the Circle of Willis has great significance for collateral flow in the event of ischemic stroke and helps patients to achieve more favorable outcomes, as it determines the affected brain territory. The clinical outcome of the ischemic stroke appears to be significantly better if only one artery territory is affected, compared to two or more.

## 1. What This Research Adds

### 1.1. What Is Already Known on This Topic 

Multiple authors have highlighted the importance of Circle of Willis integrity for autoregulation and sufficient blood flow in cases of an occlusion. Published studies have shown that the presence of Circle of Willis branches has a direct impact on stroke outcome as well, with patients achieving statistically significantly more favorable results when certain branches of the Circle of Willis were present, especially in cases of distal internal carotid T occlusion. Thrombus location is just as important as Circle of Willis integrity, since the combination of these two factors can determine whether one or more brain territories are affected by ischemia, which in turn leads to worse outcomes. Different configurations of the Circle of Willis have varying impacts in cases of ischemic stroke, as they depend on the location of the thrombus—for example, in cases of an M1 occlusion, Circle of Willis configuration has no impact, while in cases of distal ICA occlusion and contralateral A1 aplasia, it has a profound impact, as proven by multiple studies. The aim of this study was to review the available data on the Circle of Willis configuration and thrombus location impact on patient outcome in cases of ischemic stroke.

### 1.2. What This Study Adds 

This study highlights the great significance of the integrity of the Circle of Willis and location of the thrombus for collateral flow in the event of ischemic stroke, as two of these factors have a direct influence on the affected brain territory and potential patient outcomes. We reviewed the literature and summarized the different Circle of Willis variants together with possible thrombus locations and pointed out how the combination of two of these factors has different impacts within distinct variants. To our knowledge, this is the first literature review on this topic; therefore, it can serve as a concise summary of the available data. This study concisely explains which variants are important in certain levels of occlusion and in what way. We also point out the Circle of Willis configuration and thrombus location pairs that have very limited data and could be an area of future research.

### 1.3. How This Study might Affect Research, Practice, or Policy

This study summarizes the available literature on Circle of Willis integrity and the thrombus location impact of ischemic stroke outcomes and can be used as a reference for practice in certain ischemic stroke patients, as we analyzed which Circle of Willis variants and thrombus locations have an impact on patient outcomes. We also point out which configurations have statistically worse outcomes, based on the available literature. Certain Circle of Willis variants and thrombus locations are not analyzed in the available literature—this could be an area of future research. We also note that the description of Circle of Willis integrity and hypoplasia varies across studies. A unified description/classification system for accurate comparison between different studies is needed and would allow for a bigger cohort analysis.

## 2. Background

Ischemic brain stroke accounts for 70% of all strokes and is the third most common cause of death and a major contributor to disability, therefore inflicting a severe economic burden [1]. The prompt and precise treatment of stroke is essential, as number of irreversibly changed brain territory increases with time [2]. Knowledge of the patients’ cerebrovascular anatomy needs to be considered when treating a stroke, as an incomplete Circle of Willis has been linked to worse stroke outcomes by some authors [3]. Certain anatomical variants could be a potential prompt for a more invasive treatment, e.g., when ischemic changes occur in a large brain territory due to a specific configuration of Circle of Willis.

The general population has considerable variations in Circle of Willis morphology, with studies reporting only about half of patients having a complete Circle of Willis [4]. A few published studies suggest that the prevalence of the incomplete Circle of Willis is higher in patients with ischemic stroke compared to healthy controls and varies from 34% to 82.4%; however, recent meta-analysis did not find an association between incomplete CoW and ischemic stroke occurrence [3,4,5,6]. 

Multiple authors have highlighted the importance of the Circle of Willis integrity for autoregulation and sufficient blood flow in cases of occlusion [6,7,8]. Despite autoregulation being an important factor in ensuring sufficient blood flow (>90% normal flow) in cases of internal carotid artery occlusion, it alone cannot provide adequate blood flow [8]. In cases of an occlusion, integrity of the Circle of Willis becomes even more important. Published studies have shown that the presence of communicating arteries has a direct impact on stroke outcome, with patients achieving more favorable results when communicating arteries were present, especially in cases of distal internal carotid T occlusion [7].

In cases of an ischemic stroke, it is important to consider the patients’ variant of Circle of Willis in relation to the thrombus localization, as it can have a significant effect on the outcome due to a larger affected territory, e.g., distal ICA occlusion with contralateral A1 aplasia would cause ischemia in ipsilateral MCA and ACA territories, instead of only MCA territory when contralateral A1 is patent [9].

The aim of this study was to review the available data on the Circle of Willis configuration and thrombus location impact on patient outcome in cases of ischemic stroke.

## 3. Methods

1. Literature search strategy

Three authors independently performed a literature search to identify studies that investigate the influence of Circle of Willis variants on stroke outcomes. PubMed database was searched for manuscripts published up to March 2023 using the following keywords: “Stroke” [MeSH] AND “Circle of Willis” [MeSH] AND “Variants” [MeSH]. Free text words were also used to avoid missing recent manuscripts that had not yet been given a Mesh label. A total of 67 studies were found, and 11 studies were deemed eligible after reading the abstracts. 

The EMBASE database was also checked for relevant studies, published up to March 2023, using the following keywords: “Stroke” [MeSH] AND “Circle of Willis” [MeSH] AND “Variants” [MeSH]. A total of 90 studies were found, and 5 were eligible. 

The Cochrane Database of Systematic Reviews was searched using the following words: “Stroke”, “Circle of Willis”, and “Variants”. No reviews were found.

Any disagreements the authors had were resolved through comprehensive discussion after independent readings of the full text. 

**Figure 1:**
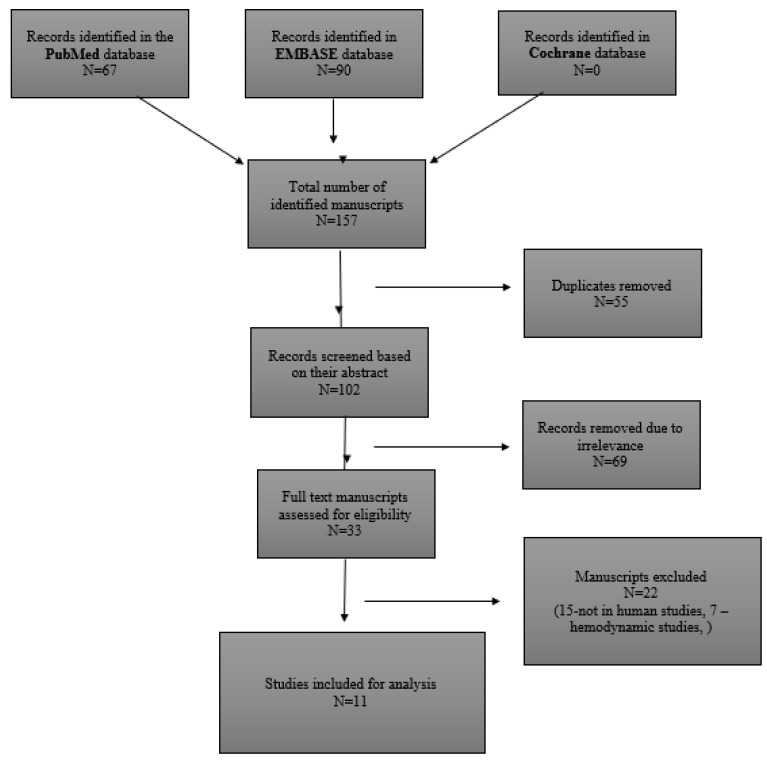
Literature search strategy and outcomes.

The full approach to the literature search and article selection according to PRISMA guidelines is outlined in Figure 1.

2. Selection criteria

Inclusion Criteria● publications on the influence of Circle of Willis variants on ischemic stroke outcomes;● manuscripts in English, German, and Russian;● human studies;● available in full text. Exclusion criteria● hemodynamic model studies;● only abstract available;● physiology reviews;● case-series and case reports.

3. Types of studies human studies;
4. Types of participants: ischemic stroke patients;
5. Types of outcomes.

The outcome measure was defined as the clinical relevance Circle of Willis variants in ischemic stroke patients and their outcomes.

6. Data extraction and critical appraisal

After identifying relevant titles, all abstracts were read independently, and full text manuscripts were accessed through a Vilnius university VPN. A manual cross-reference search of references of relevant manuscripts was also performed identify other studies that were not selected in the previous search. The methodological validity assessment of each manuscript was performed using the methodological index for nonrandomized studies (MINORS) quality score [10]. Non-comparative studies were evaluated on eight quality items, while comparative studies were evaluated on on twelve quality items. For each quality item, a score of 0 indicates that it was not reported in the manuscript, 1 indicates that it was reported but inadequately, and 2 indicates that it was reported adequately. This sums up to a maximum MINORS score of 16 for non-comparative studies and 24 for comparative studies. In this review, a score of ≤6 was considered poor quality. Quality assessment results of the studies included are demonstrated in Table 1. The systemic review of studies was performed using the PRISMA guidelines [11].

## 4. Results

This systematic review analyzed 11 studies (*n* = 4643) on the impact of Circle of Willis on stroke outcomes. Upon the systematic literature review, two major different study groups were identified: studies with identified occlusion level and specified treatment (either MTE or IVT) and those with no identified occlusion level and no treatment specified. 

Distal ICA and M1 occlusions were most prevalent in studies with identified occlusion levels. Studies with distal ICA, M1, or A1 occlusions had the highest median National Institute of Health Stroke Scale (NIHSS) scores in our analyzed studies (Table 2).

## 5. Discussion

### 5.1. Importance of Circle of Willis Variants for Stroke Outcomes

Data on the impact of Circle of Willis variants on stroke outcome are contradictory across published studies. The integrity of Willis’ Circle is strongly associated with stroke severity, hinting that having a complete Willis’ Circle has some kind of protective effect in an acute ischemic stroke [7,12]. Lin et at. found that the presence of an incomplete circle of Willis decreased the odds of a stroke patient having a good outcome by 47%, after adjusting for age and severity of stroke at admission, while the study by Westphal et al. did not confirm these findings—CoW integrity was not different in groups with favorable (0–2 mRS) and unfavorable (3–6 mRS) 3-month outcomes, However, a trend towards higher mortality in patients with any type of CoW variants was found [4,5]. 

The importance of collateral blood supply was highlighted by Zhao et al.—in the primary collateral grade group with present anterior and posterior communicating arteries, 61.5% of the patients achieved favorable outcomes, while only 37.5% of those in primary collateral grade groups without either anterior or posterior communicating arteries did so [7]. A strong limitation of this study is the fact that all the configurations of CoW were compared to each other simultaneously, and therefore it is not possible to tell which specific configuration has the most impact on the stroke outcome, but an assumption can be made that the presence of communicating arteries increases the chance of a favorable outcome after ischemic stroke. 

### 5.2. Distal ICA Occlusion

Several studies that included mechanical thrombectomy patients and reported occlusion sites highlighted the importance of A1 segments’ role in the setting of distal ICA occlusion. The studies by Lee et al. and Fischer et al. reported that patients with hypoplastic or absent contralateral A1 had significantly worse NIHSS at discharge and 3-month mRS scores, while in the study by Seilfert-Held et al., where ipsilateral hypoplastic A1 segments were analyzed, results between groups were similar [13,14,16]. 

A study by Seilfert-Held et al. analyzed the impact of ipsilateral CoW variants in distal ICA or MCA occlusion. The study included the ipsilateral posterior communicating artery, ipsilateral P1, and ipsilateral A1 segments, but did not analyze each segment separately. No statistically significant stroke outcome differences based on the integrity of the ipsilateral part of the Circle of Willis were found. 

**Figure 2:**
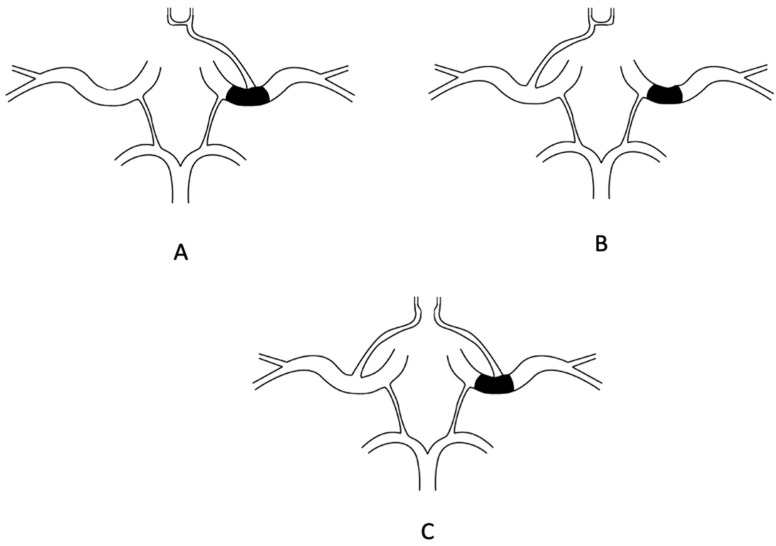
(**A**): Distal ICA occlusion with aplastic contralateral A1 segment. Perfusion is compromised in ipsilateral MCA and both ACA territories. Perfusion is compromised in contralateral ACA since blood on the ipsilateral side has to travel via leptomeningeal collaterals from contralateral ICA to contralateral ACA and then to ipsilateral ACA. This is an example of a three-territory ischemic stroke. (**B**): Distal ICA occlusion with aplastic ipsilateral A1 segment. In this case, perfusion is compromised in ipsilateral MCA territory, but ipsilateral ACA pool is perfused via anterior communicating artery from the contralateral side. This is classified as a one-territory ischemic stroke. (**C**): Aplasia of anterior communicating artery in cases of distal ICA occlusion. Perfusion is compromised in ipsilateral MCA and ACA territories. An example of a two-territory ischemic stroke.

Contralateral A1 aplasia in the case of an ICA occlusion was analyzed by Fisher et al. Contralateral A1 aplasia is critically important, as the protective collateral blood supply from the Circle of Willis via the anterior communicating artery is compromised. When cA1 is absent or occluded, there is no antegrade filling of both ACA territories and subsequently no further collateral circulation through an opening of anastomoses between the ACAs and the occluded MCA [7]. With this CoW variant, distal ICA occlusion leads to ischemic changes in ipsilateral MCA and both ACA territories (Figure 2A), instead of only the ipsilateral MCA territory (Figure 2B). 

In cases of anterior communicating artery aplasia with distal ICA occlusion, ischemic changes occur in ipsilateral MCA and ACA (Figure 2C). A study by Zhao et al. found that 94.11% of the patients with an absent anterior communicating artery achieved unfavorable outcomes, compared with 5.88% with favorable outcomes [7]. With anterior communicating artery present, outcomes were 62.5% and 37.5%, respectively.

### 5.3. Fetal Posterior Cerebral Artery

Studies that evaluated the impact of the presence of posterior communicating artery on stroke outcome did not find a statistically significant difference in NIHSS on admission or early stroke outcomes [7,15,17]. This could be due to the fact that the location of thrombus was not evaluated together with aplasia/hypoplasia of posterior communicating artery, as this can have an impact on the size of ischemic zone. 

As pointed out by Hong et al., in some cases, fetal PCA can have a positive effect on the stroke outcome—in cases of acute stroke involving the brainstem with BA occlusive disease, fetal PCA was found as an independent predictor of good prognosis. This result can be explained by the fact that patients with fetal PCA have a smaller area of posterior circulation; therefore, in cases of BA occlusion, relatively little brain stem perfusion is lost, and the possibility of retrograde filling into the upper brainstem through the fetal-variant PcoA prevails [18].

### 5.4. Circle of Willis Variants Has No Impact in Cases of M1 Occlusion

As MCA M1 segment is distal to the Circle of Willis, its anatomical variants should have no impact in the case of M1 occlusion. Westphal et al. conducted a study that found that in the event of M1 occlusion, CoW integrity did not have an impact on NIHSS on admission and 3-month outcome, measured using a modified Rankin Scale. The authors also did not find statistically significant differences in CoW variants with M1 ischemic stroke [5].

## 6. Conclusions

The preserved integrity of the Circle of Willis has great significance for collateral flow in the event of ischemic stroke and helps patients to achieve more favorable outcomes, as it determines the affected brain territory. The clinical outcome of the ischemic stroke appears to be significantly better when only one artery territory is affected, compared to two or more. In cases of distal ICA occlusion with contralateral A1 aplasia, stroke outcomes were statistically significantly worse than without aplasia, as three of the brain territories are affected by stroke.

We presume that in the event of distal internal carotid artery occlusion, the presence of a fully developed contralateral A1 segment and anterior communicating artery is essential for a favorable stroke outcome. Circle of Willis integrity plays a vital role in stroke outcome, especially when T-form occlusions are present. Despite this, in the event of M1 occlusion, Circle of Willis configuration does not play an important role. The impact of posterior communicating artery integrity in cases of an occlusion has controversial results in the literature; therefore, further studies need to be conducted.

It is important to evaluate the level of occlusion together with the integrity of the Circle of Willis since the combination of these factors has an impact on the predicted outcome of the patient, which is a beneficial direction for future research.

Further studies regarding Circle of Willis configuration and thrombus location impact on stroke outcomes need to be conducted. The definition of Circle of Willis integrity across studies should become more uniform, as this would allow for more comparable studies and better data for meta-analysis, potentially yielding new insights in stroke treatment. The complete anterior part of the Circle of Willis could be treated as having the presence of all branches—both M1 and A1 and the anterior communicating artery. Correspondingly, the posterior part could be treated as a whole when both P1 and the posterior communicating arteries are present. The definition of hypoplasia should be unified, treating an artery with an external diameter of less than 1 mm hypoplastic as well as a communicating artery of external diameter of less than 0.5 mm hypoplastic [19].

## 7. Limitations

The description of Circle of Willis integrity and hypoplasia varies across studies. A unified description/classification system for the accurate comparison between different studies is lacking and would allow for a bigger cohort analysis, therefore yielding more statistically reliable results. The number of studies regarding the variants of the whole Circle of Willis anatomy and the localization of the thrombus impact on stroke outcome is very limited as well.

## Figures and Tables

**Table 1:** Quality assessment of the included studies.

Study	A Clearly Stated Aim	Inclusion of Consecutive Patients	Prospective Collection of Data	Endpoints Appropriate to the Aim of the Study	Unbiased Assessment of the Study Endpoint	Follow-Up Period Appropriate to the Aim of the Study	Loss to Follow Up Less than 5%	Prospective Calculation of the Study Size	An Adequate Control Group	Contemporary Groups	Baseline Equivalence of Groups	Adequate Statistical Analyses	MINORS
De Caro et al. [4]	2	2	0	2	2	n/a	n/a	0	2	2	2	2	16
Lin et al. [1]	2	2	0	2	2	2	2	0	2	2	2	2	20
Westphal et al. [2]	2	2	0	2	2	2	0	0	n/a	n/a	n/a	2	12
Fischer et al. [12]	2	2	0	2	1	2	1	0	n/a	n/a	n/a	n/a	10
Seifert-Held et al. [13]	2	0	0	2	2	2	0	0	n/a	n/a	n/a	1	9
Shaban et al. [14]	2	0	0	2	2	2	0	0	n/a	n/a	n/a	2	10
Hong et al. [15]	2	2	1	2	2	0	0	0	n/a	n/a	n/a	n/a	9
Zhao et al. [5]	2	2	0	2	1	2	1	0	n/a	n/a	n/a	n/a	10
Lee et al. [11]	2	2	0	2	2	2	1	0	n/a	n/a	n/a	n/a	11
Zhou et al. [10]	1	2	1	2	2	1	0	0	n/a	n/a	n/a	n/a	9

**Table 2:** Summary of included studies.

Study	Country	Study Design	N	Occlusion Site	NIHSS on Arrival	CoW Variants	Stroke Territory	Treatment	Results	Conclusion	Link	MINORS
De Caro et al. (2021) [4]	Italy	Cross-sectional study	1131 + 562 control	Not documented	11.4 ± 7.1	1. No variants 2. Ant. variants 3. Post. Variants 4. Ant. and post. variants	All	MTE ± IVT	Among stroke patients, 702 (62.1%) had one or more vascular variants, compared to 308 (54.8%) of the control group (*p* < 0.01), 165/702 (23.5%) had an anterior circulation variant only, 384/702 (54.7%) had a posterior circulation variant only, and 153/702 (21.8%) patients had variants in both anterior and posterior circulation.	No statistical significance	https://pubmed.ncbi.nlm.nih.gov/33786665/ (accessed on 11 June 2023)	16/24
Lin et al. (2022) [1]	USA	Cross-sectional study	297	43% had an intracranial occlusion, not detailed	5.0 ± 7.2	P1, A1	N/a	Not documented	The presence of an incomplete circle of Willis decreased the odds of a stroke patient having a good outcome by 47% (*p* = 0.046, OR 0.53, 95% CI 0.281–0.988), after adjusting for age and severity of stroke at admission.	An incomplete circle of Willis may be associated with a poorer prognosis for stroke patients.	https://pubmed.ncbi.nlm.nih.gov/35661459/ (accessed on 11 June 2023)	20/24
Westphal et al. (2021) [2]	Switzerland	Cross-sectional study	193 + 73	M1	12 (8–17)	Four flow models (A1/AcomA, P1/PcomA)	M1	MTE ± IVT	CoW integrity did not differ between groups with favorable (modified Rankin Scale (mRS)): 0–2) and unfavorable (mRS: 3–6) 3-month outcome. However, trends towards a higher mortality in patients with any type of CoW variant (*p* = 0.08) and a higher frequency of incomplete CoW among patients dying within 3 months after stroke onset (*p* = 0.119) were found. In a logistic regression analysis adjusted for the potential confounders age, sex, and atrial fibrillation, neither the vascular models nor anterior or posterior variants were independently associated with outcome.	No statistical significance	https://pubmed.ncbi.nlm.nih.gov/34233384/ (accessed on 11 June 2023)	13/16
Seifert-Held et al. (2021) [13]	Austria	Cross-sectional study	182	ICA dist., MCA	16	Absence of either the iPcomA, iP1 or iA1 were all rated as an incomplete CoW.	Anterior	MTE ± IVT	An incomplete ipsilateral COW was not predictive of the patients’ functional outcome at 90 days.	No statistical significance	https://pubmed.ncbi.nlm.nih.gov/34233384/ (accessed on 11 June 2023)	9/16
Shaban et al. (2013) [14]	USA	Case control	536	Not documented	7 (0–27) 3, 14	cfPCA/pfPCA/nofPCA	All	Not documented	Patients with complete fPCA had fewer small vessel strokes and more large vessel strokes than patients with no fPCA and partial fPCA. Fetal PCA may predispose an individual toward stroke mechanism, but it is not associated with vascular distribution, stroke severity, or early outcome.	No statistical significance	https://pubmed.ncbi.nlm.nih.gov/23577277/ (accessed on 11 June 2023)	9/16
Hong et al. (2009) [15]	South Korea	Case control	95	PA	10.4 (9.7)	fetal vs. non-fetal PcomA	VB	Natural progression	Among all 95 patients, 58% (n = 55) had good prognosis (mRS (2). Interestingly, 44 patients (46.3%) had at least one fetal-variant PcomA (26 bilateral, 18 unilateral). Through multiple logistic regression analysis, the atherosclerotic mechanism (OR 18.0; 95% CI 3.0 to 107.0) and presence of fetal-variant PcomA (OR 5.1; 95% CI 1.4 to 18.8) were found to be independent predictors for good prognosis and initial NIH stroke scale score (OR 1.24 per one-point increase; 95% CI 1.1 to 1.4) for poor prognosis.	Fetal PcomA appears to be an important factor for positive outcomes in acute stroke victims involving the brainstem with BA occlusive disease	https://pubmed.ncbi.nlm.nih.gov/19917819/ (accessed on 11 June 2023)	10/16
Zhao et al. (2019) [5]	China	Cohort study	38	PcomA + T or isolated PcomA	23	1. AcomA (−) 2. AcomA (+); iPcomA (−) 3. AcomA (+); iPcomA (+)	Intracranial ICA	MTE ± IVT	Of 38 iICAO patients, 65.8% (25 in 38) achieved reperfusion. However, only 31.6% (12/38) achieved favorable outcomes at 90 days. With a PCG3, 61.5% of them achieved favorable outcomes, while only 37.5% of those with PCG2 and PCG1 achieved favorable outcomes (*p* = 0.003). In multivariable logistic regression, PCG was revealed as a predictor for favorable outcomes (OR 5.278, *p* = 0.019) after adjusting the reperfusion and other factors.	The PCG based on the integrity of Willis’ Circle might be an underlying predictor of the prognosis of AIS in patients with iICAO after MTE.	https://www.ncbi.nlm.nih.gov/pmc/articles/PMC6908856/ (accessed on 11 June 2023)	10/16
Fischer et al. (2022) [12]	Germany	Case control	1068	Intracranial ICA	17 (13–21)	Normal A1 vs. functional aplasia	Intracranial ICA	MTE ± IVT	Patients with functional contralateral A1 aplasia were more severely affected on admission (median NIHSS 18, IQR 15–23 vs. 17, IQR 13–21; aOR: 0.672, 95% CI: 0.448–1.007, *p* = 0.054) and post-interventional ischemic damage was larger (median ASPECTS 5, IQR 1–7, vs. 6, IQR 3–8; aOR: 1.817, 95% CI: 1.184–2.789, *p* = 0.006). Infarction occurred more often within the ipsilateral ACA territory (20/76, 26% vs. 110/961, 11%; aOR: 2.482, 95% CI: 1.389–4.437, *p* = 0.002) and both ACA territories (8/76, 11% vs. 5/961, 1%; aOR: 17.968, 95% CI: 4.979–64.847, *p* ≤ 0.001). Functional contralateral A1 aplasia was associated with a lower rate of functional independence at discharge (6/81, 8% vs. 194/965, 20%; aOR: 2.579, 95% CI: 1.086–6.122, *p* = 0.032) and after 90 days (5/55, 9% vs. 170/723, 24%; aOR: 2.664, 95% CI: 1.031–6.883, *p* = 0.043).	A functional A1 aplasia contralateral to a distal ICA occlusion associated with a poorer clinical outcome	https://www.mdpi.com/2077-0383/11/5/1293 (accessed on 11 June 2023)	11/16
Lee et al. (2016) [11]	South Korea	Cohort study	92	T or L type occlusions	19 (17–22)	The STO group was classified by acute ICA terminus occlusion and patency of not only the ipsilateral A2 segment via the AcomA but also the ipsilateral PCA via the PcomA or the P1 segment. The CTO group was classified by acute ICA terminus occlusion and had one or more of the following: 1) occlusion of the ipsilateral A2 or more distal segment 2) occlusion of the fetal-type ipsilateral PCA (C6 segment of ICA) 3) insufficient contralateral Willisian collateral blood supply via the AcomA due to contralateral agenesis of A1 (absent or severely hypoplastic) contralateral ICA occlusion.	Anterior	IVT ± MTE,	The STO group (n = 58) showed smaller infarct volumes at 72 h than the CTO group (n = 34) (median, 81 mL (interquartile range, 38–192) vs. (414 mL 193–540, *p* < 0.001) and more favorable outcomes (3-month modified Rankin Scale 0–3, 44.8% vs. 8.8%, *p* < 0.001; 3-month mortality, 24.1% vs. 67.6%, *p* < 0.001). In multivariable analyses, STO remained an independent predictor for favorable outcomes (odds ratio 6.1, *p* = 0.010).	The acute ICA terminus occlusion outcomes depend on Willisian collateral status.	https://www.ncbi.nlm.nih.gov/pmc/articles/PMC4901942/ (accessed on 11 June 2023)	9/16
Zhou et al. (2016) [10]	China	Cohort study	376	Not documented	7.79 ± 5.16	Four CoW variants: PCG1: complete Circle of Willis; type PCG2: complete anterior half of the circle of Willis and incomplete posterior half of the circle of Willis; PCG3: incomplete anterior half of the circle of Willis and complete posterior half of the circle of Willis; and type PCG4: incomplete anterior and posterior halves of the circle of Willis.	Not documented	Natural progression	Of 38 iICAO patients, 65.8% (25 in 38) achieved reperfusion. However, only 31.6% (12/38) achieved favorable outcomes after 90 days. With a PCG3, 61.5% of them achieved favorable outcomes, while only 37.5% of those with PCG2 and PCG1 achieved favorable outcomes (*p* = 0.003). In multivariable logistic regression, PCG was revealed as a predictor for favorable outcomes (OR 5.278, *p* = 0.019) after adjusting the reperfusion and other factors.	Better functional outcomes in patients with complete CoW	https://pubmed.ncbi.nlm.nih.gov/26962785/ (accessed on 11 June 2023)	10/16

MTE—endovascular mechanical thrombectomy; IVT—intravenous thrombolysis; cfPCA—complete fetal posterior cerebral artery; pfPCA—partial fetal posterior cerebral artery; nofPCA—no fetal posterior cerebral artery; fPCA—fetal posterior cerebral artery; iPcomA—ipsilateral posterior communicating artery; AcomA—anterior communicating artery; iICAO—internal carotid artery occlusion; STO—simple ICA terminus occlusion; CTO—complex ICA terminus occlusion; ICA—internal carotid artery; AIS—acute ischemic stroke.

## Data Availability

No new data were used; all the reviewed data can be found in the respective research articles in the references.
